# ﻿Type specimens, taxonomic history, and genetic analysis of the Japanese dancing mouse or waltzer, *Muswagneri* variety *rotans* Droogleever Fortuyn, 1912 (Mammalia, Muridae)

**DOI:** 10.3897/zookeys.1200.118823

**Published:** 2024-05-02

**Authors:** Mónica Cruz, Wim Bergmans, Toyoyuki Takada, Toshihiko Shiroishi, Atsushi Yoshiki

**Affiliations:** 1 Naturalis Biodiversity Center, Darwinweg 2, 2333 CR Leiden, Netherlands; 2 RIKEN BioResourse Research Center, Tsukuba Ibaraki 305-0074, Japan; † Deceased

**Keywords:** Crossbred, genetic analysis, genome, Japanese dancing mouse, lectotype, taxonomic position

## Abstract

In the present paper, the existence and location of the type series of the Japanese dancing mouse or waltzer, *Muswagneri* variety *rotans* Droogleever Fortuyn, 1912, are established, and a lectotype is designated. Available type specimens are measured, and some morphological parameters, sex, and general condition of the specimens are recorded. A literature survey was conducted, and an attempt is made to clarify the position of *M.wagneri* variety *rotans* in the taxonomy of *Mus.* A genetic analysis suggests that the type series of the Japanese dancing mouse represent a crossbred, or derivation of a crossbred, between the original Japanese dancing mouse of *Musmusculusmolossinus* Temminck 1844 origin and European fancy or laboratory mice of *Musmusculusdomesticus* Schwarz & Schwarz, 1943 origin. Much of their genome was replaced and occupied by *Musmusculusdomesticus* type genome, probably through extensive breeding with European mice.

## ﻿Introduction

In 1912 Æ.B. Droogleever Fortuyn, a Dutch scientist who worked mainly on the anatomy and the heredity of traits in the common house mouse, *Musmusculus* Linnaeus, 1758, and related taxa, described *Muswagneri* variety *rotans*, the Japanese dancing mouse, a form owing its name to its peculiar rotatory movements. In the fancy mice literature, it is often referred to as waltzing mice, or waltzers. He based his description on 11 specimens, 10 of which were imported from Vienna, Austria, by Dr. C. Kerbert, the director of the Royal Zoological Society Natura Artis Magistra in Amsterdam. The remaining specimen was bred in a laboratory in Utrecht by prof. Dr Zwaardemaker and made available to Droogleever Fortuyn by Dr C.U.A. Kappers (Droogleever Fortuyn 1912). In his description, Droogleever Fortuyn did not designate a holotype. He also did not state where the type specimens were deposited.

During routine curating activities by the second author in the Mammal Department of the former Zoological Museum of the University of Amsterdam (ZMA; now incorporated in Naturalis Biodiversity Center in Leiden, the Netherlands), a glass jar with piebald mice was found labelled (slashes added): “G / Typen / *Muswagneri* varietas *rotans* Droogl. Fort. / Japansche Dansmuis”. The jar contained 10 specimens of Japanese dancing mice preserved in 70% ethanol. Given the fact that the specimens are labelled “typen” (= types) there can be no doubt that they-together with the specimen from Utrecht, which has not been located-constituted the basis for Droogleever Fortuyn’s description and that they are the type specimens of *M.wagneri* variety *rotans* Droogleever Fortuyn, 1912. The “G” on the label indicates that at the time of their description the type specimens formed part of the zoological collection of Amsterdam’s municipality (Dutch: gemeente). Droogleever Fortuyn based his description on several measurements of these specimens. In addition, he found that, in comparison with *M.musculus*, the specimens had fewer tail rings (Droogleever Fortuyn 1912). Apart from the fact that they are type material, the specimens are highly valuable to elucidate the origin of common laboratory mouse strains.

When Droogleever Fortuyn described the Japanese dancing mouse in 1912, *Muswagneri* Eversmann, 1848 was considered a full species. Its low tail ring number constituted one of the characters used to distinguish it from the otherwise closely related *M.musculus*. Due to this, and to the fact that *M.wagneri* was the only Asian wild mouse species known to Droogleever Fortuyn (Schwarz 1942), he was convinced that the Japanese dancing mouse was a form of *M.wagneri* characterized by abnormal spinning or rotatory movements.

**Figure 1.**
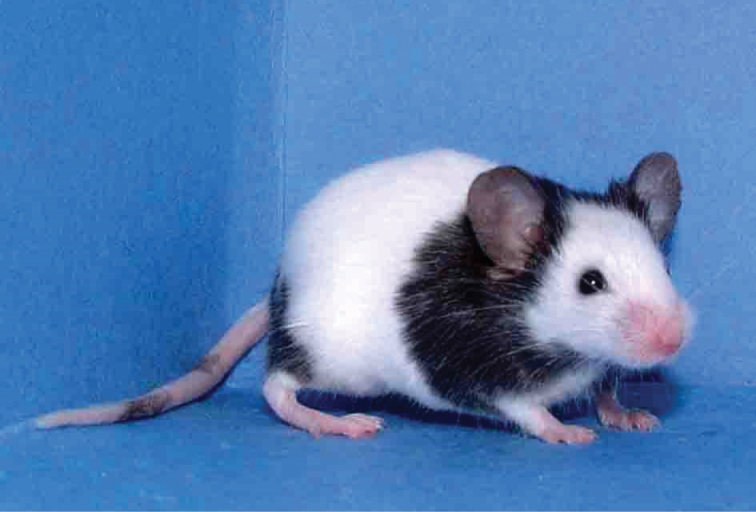
The JF1 mouse strain has been developed at the National Institute of Genetics in Mishima, Japan, and is available for distribution to biomedical researchers from the RIKEN BioResource Center in Tsukuba, Japan.

The Japanese have long nurtured the tradition of keeping and breeding mice as pets. The varieties of mice kept and bred by Japanese collectors (or “fanciers”) have been known through the centuries as “fancy mice” and include agouti, albino, and piebald fur colours, pink-eyed dilution, and dwarf-built (Koide et al. 1998), as well as Japanese dancing mice, which in the fancy mice literature are often referred to as waltzing mice, or waltzers. The coat colour pattern of the present specimens is typical non-agouti and piebald, highly resembling that of the Japanese Fancy Mouse 1 (JF1) inbred strain (Koide et al. 1998). The JF1 strain has been established from a pair of mice with non-agouti and piebald coat colour kept as Japanese Mice at a market in Denmark. Similar fancy mice with non-agouti piebald coat colour were described in the Japanese literature at the end of the 1700s, suggesting ancestors of JF1 mice were transferred overseas. Moreover, the Japanese dancing mice or Japanese waltzers are known to have contributed in the early stage of establishment of laboratory mouse strains widely used in biomedical studies (Keeler 1931; Morse 1978). Later, it was proven by whole genome sequencing of JF1 mice and genome comparison with a classical inbred laboratory strain C57BL/6J (Takada et al. 2013). Fig. 1 shows a Japanese dancing mouse, and video showing both fur colour and rotatory movements thought to resemble those of the Japanese dancing mouse can be found on the Internet (http://www.youtube.com/watch?v=hmMfAvxyBh4).

The aims of the present study are to trace the history of the Japanese dancing mouse, *Muswagneri* variety *rotans* Droogleever Fortuyn, 1912, in taxonomy, to establish the existence and location of the types and to designate a lectotype, to give descriptive notes, and to present a genetic analysis of the type material to define their genetic status in comparison with JF1 mice.

## ﻿Materials and methods

### ﻿Materials

Lectotype, 1 male, ZMA.MAM.27233, in 70% ethanol; a laboratory animal preserved in 1912 by Æ.B. Droogleever Fortuyn in Amsterdam, no further data.

Paralectotypes, 5 males, 3 females, 1 cf. female, ZMA.MAM.27234-27242, same data. All specimens are now incorporated in the collections of the Naturalis Biodiversity Center in Leiden (NBC). They were individually labelled by Droogleever Fortuyn with Greek letters. Prior to their inclusion in the ZMA collections, the specimens formed part of the natural history collection of Amsterdam’s municipality.

### ﻿Methods

All specimens were measured, and some morphological parameters, sex, and general condition were recorded. In addition, a lectotype was designated. A literature survey was conducted, and an attempt was made to clarify the taxonomic position of *Muswagneri* variety *rotans* in *Mus.* Three specimens, registered as ZMA.MAM.27233, 27239, and 27241, were used for a genetic analysis. A 5 × 5 mm-square skin fragment of each of these specimens was dissected and processed using the DNA extraction kit (QIAGEN) to obtain genomic DNA for genotyping analyses. Genotyping was performed with a panel of 95 simple sequence length polymorphism markers, which can distinguish C57BL/6J and JF1 mouse strains (Kikkawa et al. 2001; Takada et al. 2008). Single nucleotide polymorphism (SNP) genotyping was carried out for 977 SNP marker loci which had been found to be polymorphic between MSM, belonging to the same substrain as JF1 and C57BL/6J in a previous study (Takada et al. 2013) using the MassARRAY iPLEX system (Sequenom Inc., San Diego, USA). The SNP information is also available at MoG+ (Mouse Genome variation database) (Takada et al. 2021), https://molossinus.brc.riken.jp/mogplus/#JF1). The data were recorded and interpreted using MassARRAY software (Sequenom Inc.). Analyses were repeated twice and only reproduced results were counted.

Genomic DNA extracted by a standard method from JF1 and MSM as controls of *Musmusculusmolossinus*-origin subspecies, and that of C57BL/6J mice as controls of *M.musculusdomesticus*-origin subspecies, were used to compare the genotype with the ZMA series.

### ﻿Definitions and abbreviations

**BT** broken tail

**CB** condylobasal length: distance between anterior face of incisor or anterior tip of nasal bones (depending on which is more anterior) and posterior face of occipital condyle

**E** ear length

**HB** head and body length: distance between tip of snout and anus

**HF** hind foot length: distance between tip of longest digit excluding claw and posterior tip of heel

**ID** individual identification on original label

**JF1** Japanese fancy mouse 1

**MR** molar row length: distance between anterior rim of M^1^ alveolus and posterior rim of M^3^ alveolus

**MSM** an inbred strain established from Japanese wild mice, M. m. molossinus, collected in 1978 in Mishima, Shizuoka-ken

**NIG** National Institute of Genetics, Mishima, Japan

**S** sex

**SC** skull crushed: skull severely damaged; no skull measurements can be taken

**SD** skull damaged: not all measurements can be taken

**Sd** standard deviation

**SI** skull intact

**SO** skin opening on head

**T** tail length: distance between anus and tip of tail, excluding terminal hair tip

**TL** total length: distance between tip of snout and tip of tail, excluding terminal hair tip

**ZB** zygomatic breadth: distance across most distal points of zygomata

**ZMA** Zoological Museum of the University of Amsterdam

**ZMAcd** code number in former ZMA database (now incorporated in the database of Naturalis Biodiversity Center; all numbers now have a new prefix: ZMA.MAM.)

## ﻿Results

### ﻿Origin and taxonomic history of the Japanese dancing mouse

Japanese fancy mice are mentioned in the literature before 1800. In the Edo era (1603–1868) fancy mice were very popular in Japan and were bred as a hobby (Yoshiki and Moriwaki 2006). Artists such as Hokusai Katsushika and Kyōsai Kawanabe used fancy mice as subjects in their drawings (Yoshiki and Moriwaki 2006). The booklet “Chingan Sodategusa”, translated as “How to breed fancy mice”, was published by Chobe Zeniya in Kyoto in 1787 (Yoshiki and Moriwaki 2006). The earliest scientific record pertaining to the origin of the dancing mouse was found in the work of Yerkes (1907), who was unable to find mention of the animal in the scientific literature before 1890. After consulting several Japanese and European sources he concluded that these mice originated in China and were imported to Japan where they were bred as pets. From there they were brought to Europe and America and bred as pets and as laboratory animals for studies of physiology, anatomy, and heredity. According to Yerkes (1907: 15), “historical research indicates that a structural variation or mutation which occasionally appears in *Musmusculus*, and causes those peculiarities of movement which are known as dancing, has been preserved and accentuated through selective breeding by the Chinese and the Japanese, until finally a distinct race of mice which breeds true to the dance character has been established. The age of the race is not definitely known, but it is supposed to have existed for several centuries.”

In 1912 the Japanese dancing mouse was described, as Muswagnerivar.rotans, by Æmilius Bernardus Droogleever Fortuyn (1886–1970), a Dutch scientist who worked mainly on the anatomy and the heredity of traits in the common house mouse and related species (e.g. Droogleever Fortuyn 1928, 1929, 1931, 1934, 1935, 1939) and Droogleever Fortuyn and Meng (1937); he also focused on brain histology of rodents (Droogleever Fortuyn 1911, 1927). Droogleever Fortuyn (1912) noticed that the average number of tail rings in the Japanese dancing mouse is lower than that in *M.musculus* and similar to that in *M.wagneri*, which at the time was considered a full species. He considered the number of tail rings to be of paramount importance to distinguish the Japanese dancing mouse from *M.musculus* since the former has on average 136 tail rings versus 180 in the latter. Droogleever Fortuyn (1912) believed that this character was unrelated to the relative length of the tail (which is shorter in *M.wagnerirotans*) because he had found that young *Musmusculus* had shorter tails than adults while showing the same number of tail rings. For this reason, he assigned the Japanese dancing mouse to *M.wagneri*, the only Asian wild *Mus* species he knew, and given the fact that the Japanese dancing mouse exhibits a spinning behaviour not seen in typical *M.wagneri*, he chose to name the Japanese dancing mouse *Muswagneri* variety *rotans*. Gates (1925: 651–652) corroborated these findings: “… in all probability, the Japanese mouse, of both the waltzing and the non-waltzing form, is a derivative of *Muswagneri* and not *M.musculus*, the common house mouse. … In all body measurements, such as length of body, tail, fore foot, hind foot, skull, number of vertebrae, number of scale rings of the tail, position of posterior nares and incisor alveoli, the Japanese waltzer resembles Wagner’s mouse quite closely. … A characteristic pigmentation of the eye is apparently common to both the Japanese and Wagner’s mouse, but is not found in the house mouse. … The protein specificities, as determined by precipitin tests of both the Japanese and Wagner’s mouse, differs from that of the common fancy varieties. This indicates that the Japanese mouse is not a derivative of the ordinary races nor intimately related to them.”

Allen (1927) placed *M.wagneri* in the synonymy of *M.bactrianus* Blyth, 1846 and assigned *M.wagnerirotans* to *M.bactrianusgansuensis* Satunin, 1902 on the basis of the T/TL ratio. According to Allen (1927: 10), this form is derived from *M.bactrianustantillus*: “It seems altogether likely that some form of this Chinese mouse represents the original stock from which the tame black-and-white mouse of Japan is derived. … Droogleever Fortuyn (1912) has proposed the name *Muswagnerirotans* for the tame animal, a name which on account of the shortness of the tail-to-total-length ratio may be included in the synonymy of the race *gansuensis*.” Droogleever Fortuyn (1931, 1939) continued to use the name *M.wagneri* for the Chinese house mouse at least up to 1939.

The Japanese dancing (or waltzing) mouse was later allocated to *Musmolossinus* Temminck, 1844, a name used by Tokuda (Makino 1941: 308) to classify the Japanese wild mouse: “… this form (*Musmolossinus*) represents the sole species of the house mouse widely distributed through Hokkaido, Honsyu and Kyusyu. … There has been long known in Japan a remarkable variety of this species under domestication, including the white or spotted forms, being famous as the so-called Japanese waltzing mice. Although they have occasionally been described as a variety of *Musbactrianus* (or often designated as Muswagnerivar.rotans), the recent status of taxonomical conception shows, according to Dr Tokuda, that they are derivatives of *Musmolossinus*.” In a study of the comparative morphology of chromosomes of three species of mouse and their varieties, Makino (1941) found no difference between the chromosomes of the Japanese dancing mouse and of the wild form of *M.molossinus*. Furthermore, all crossings between *M.musculus* and *M.molossinus* produce fertile offspring with a normal sex ratio (Makino 1941). Schwarz (1942: 46) believed “the Japanese waltzer agrees in size and tail length with the Japanese commensal *Musmusculusmolossinus*”, and according to him “There is no need to suppose that it has been taken to Japan from elsewhere”. Schwarz and Schwarz (1943) lumped the Japanese wild mouse with *M.musculus* and classified it as a subspecies: *M.musculusmolossinus*.

Minezawa et al. (1981) supported the view that the Japanese wild mouse belongs to *M.musculusmolossinus*, on the basis of genetic distance and comparison of allelic composition between Japanese and Western Hemisphere populations. These findings agreed with previous morphological studies, especially on the shape of the anterior border of the zygomatic plate (Makino 1941; Marshall 1977).

Yonekawa et al. (1981) found that mice collected in the central and southern parts of Japan all had the same monomorphic type of mtDNA that was unique to *M.musculusmolossinus*, but later they realized that this mtDNA was closely related to that of *M.musculusmusculus* from Bulgaria (Yonekawa et al. 1982) and proposed that Japanese mice are not an independent subspecies but rather a “local race” of *M.musculus*.

Marshall (1998), in his turn, pleaded for the adoption of *M.musculusmanchu* Thomas, 1909 as the name for the Japanese house mouse instead of *M.molossinus*, a name he believes describes a hybrid between *M.musculusmanchu* and *M.castaneuscastaneus* Waterhouse, 1842 and, therefore, should be excluded from zoological nomenclature.

*Musmusculusmanchu* was recognized by Marshall (1998) as a subspecies after examination of all the skins and skulls of the *Musmusculus* group in the Smithsonian Institution collection, and comparison with their original descriptions. Marshall (1998) considers *rotans* to be a pet-store mutant of *M.musculusmanchu*.

Based on Yonekawa et al.’s (1994) mtDNA analysis, Carleton and Musser (2005) recognized four subspecies of the house mouse: *M.musculuscastaneus*, *M.m.domesticus*, *M.m.musculus*, and *M.m.bactrianus*. A fifth group, *gentilulus*, is recognized as a possible species. Because the type specimen of *M.molossinus* is a hybrid of two species, Carleton and Musser (2005) left it unassigned. Furthermore, Carleton and Musser (2005) placed *manchu*, *wagneri*, and *rotans* in the synonymy of *M.musculusmusculus*.

### ﻿Summarizing the taxonomic ideas on *Muswagneri*rotans

- *Muswagnerirotans* Droogleever Fortuyn, 1912 is described.
- Allen (1927) includes *rotans* in the synonymy of *M.bactrianusgansuensis* Satunin, 1902, based on the T/TL ratio.
- Tokuda (Makino 1941) includes *rotans* in the synonymy of *M.molossinus* Temminck, 1844.
- Makino (1941) finds no difference in chromosome morphology of the Japanese dancing mouse and *M.molossinus.*
- Schwarz (1942) includes the Japanese dancing mouse in the synonymy of *M.musculusmolossinus* based on size and TL.
- Marshall (1998) considered *rotans* to be a pet-store mutant of *M.musculusmanchu* Thomas, 1909, a name he believes should designate the Japanese house mouse.
- Carleton and Musser (2005) placed *rotans* in the synonymy of *M.musculusmusculus*, based on an mtDNA analysis by Yonekawa et al. (1994).

### ﻿Type series of *Muswagnerirotans*: measurements, morphological parameters, sex, and condition

**Figure 2.**
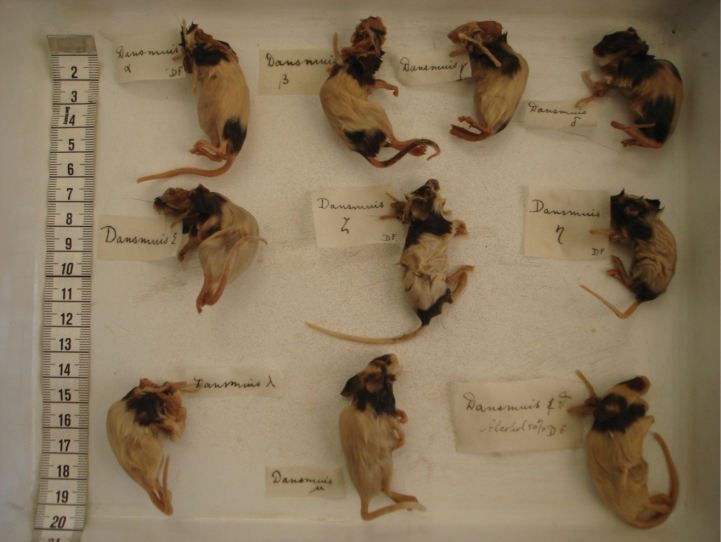
The type series of *Muswagnerirotans* Droogleever Fortuyn, 1912. From top to bottom and from left to right: Droogleever Fortuyn’s specimens α, β, γ, δ, ε, ζ, η, λ, μ, and ν (ZMA.MAM.27233–27242). Specimen μ (ZMA.MAM.27233) in the middle at the bottom is the lectotype.

The body measurements, morphological parameters, sex, and condition of all 10 type specimens and the cranial measurements of the lectotype are summarized in Table 1. HB ranges from 52.0 to 63.8 mm; T from 46.5 to 56.1 mm; HF from 10.8 to 12.7 mm, and E from 7.7 to 9.6 mm. All specimens have a black-and-white fur colour pattern (Fig. 2). The specimen labelled with the Greek letter µ (mu) was chosen as lectotype because of its generally good condition. All other type specimens are paralectotypes.

**Table 1.** Selected measurements (in mm), morphological parameters, sex, and condition of lectotype (μ; ZMA.MAM.27233) and paralectotypes (all others) of *Muswagnerirotans* Droogleever Fortuyn, 1912.

Id	ZMAcd	S	HB	T	T/HB (%)	HF	E	CB	ZB	MR	Condition
α	27234	m	58.20	46.50	79.90	11.60	8.60				SC
β	27235	m	58.30	47.20	80.96	12.55	8.70				SC
γ	27236	m	53.70			12.00	7.70				BT, SI, SO
δ	27237	f	53.90	47.30	87.76	12.65	8.40	17.20	8.00	1.20	SD, SO
ε	27238	m	59.70	54.65	91.54	12.50	9.60				SC
ζ	27239	m	56.90	51.35	90.25	12.05	8.90				SI, SO
η	27240	f	53.05	48.00	90.48	10.80	9.20				SI, SO
λ	27241	f	52.00	47.75	91.83	11.00	8.80				SC
μ	27233	m	56.10	56.10	100.00	12.70	8.90	17.50	8.00	1.40	SI
υ	27242	f (cf.)	63.80	50.50	79.15	12.45	9.50				SI
mean			56.57	49.93	87.98	12.03	8.83	17.35	8.00	1.30	
SD			3.60	3.49	6.85	0.69	0.55	0.21	0.00	0.14	

The HB variation of the specimens studied in the present work is compared with that of other *Mus* taxa in Table 2.

**Table 2.** Comparison of the HB variation (mean ± SD in mm) of the subjects of the present study (*rotans*) with the “optimum HB” of other taxa of *Mus* as given by Schwarz and Schwarz (1943).

	*M.rotans*	*M.molossinus*	*M.bactrianus*	*M.wagneri*	*M.manchu*	*M.musculus*
female	50–61	65–70	75–80	80–85	85–90	85–90
male	55–59	65–70	65–70	75–80	75–80	85–90

### ﻿Genetic analysis

Specimen ZMA.MAM.27233 was homozygous of C57BL/6-type allele in 50 out of 51 loci tested, except for one heterozygous locus on chromosome X (Table 3). This result clearly indicates that the major genomic component of the specimen was of *Musmusculusdomesticus* origin. SNP-based genotyping using the MassArray system also detected that most of the alleles of the three specimens of *M.wagnerirotans* were also of C57BL/6-type, and only 7.6, 23.2 and 37.0% of the alleles were JF1-type in ZMA.MAM.27233, 27239, and 27241, respectively.

**Table 3.** Results of genotyping of Japanese Waltzing mice. 1. B and J indicate C57BL/6J and JF1-type alleles, respectively. 2. J-type allele ratio (%) was calculated as follows: No. of loci in *B/J* + 2 × No. loci in *J/J*) / 2 × Total no. of loci successfully genotyped × 100. 3. SSLP: 95 simple sequence length polymorphism (SSLP) markers which can distinguish C57BL/6J and JF1 were used. 4. SNP: MassArray SNP analysis was conducted for 977 SNP marker loci which was known as polymorphic between C57BL/6J and JF1.

Genotyping method	Sample name	Numberof loci in each genotype^1^					
		*B/B*	*B/J*	*J/J*	Total	Not detected	J-type allete ratio^2^(%)
SSLP^3^	ZMA.MAM.27233	50	1	0	51	44	0.98
SNP^4^	ZMA.MAM.27233	840	64	40	944	33	7.6
SNP^4^	ZMA.MAM.27239	77	35	11	123	854	23.2
SNP^4^	ZMA.MAM.27241	74	17	40	131	846	37.0

## ﻿Discussion

### ﻿The place of *Muswagnerirotans* in the taxonomy of *Mus*

When comparing HB variation (mean ± SD) in *Muswagnerirotans* with the “optimum HB” of related taxa given by Schwarz and Schwarz in 1943, the most obvious observation is that *rotans* is considerably smaller than all the others (Table 2). Also noteworthy is the fact that the HB optimum of *molossinus* is “closest to *rotans*”.

Moriwaki found pairs of original fancy mice at a market in Denmark in 1987 and introduced them into the animal facility of the National Institute of Genetics (NIG) in Mishima, Japan (1998). By the 20^th^ generation resulting from sister-brother matings, a new inbred strain of Japanese fancy mouse called JF1 was established in 1993 (Koide et al. 1998). The JF1 strain carries a “spotting phenotype on the coat resembling an old mutation piebald” and is phenotypically similar to the Japanese dancing mice described by Gates (1926) and by other authors in the early 1900s (Koide et al. 1998). The phenotypical similarity of the *M.w.rotans* types as described in the present paper with the mice belonging to the JF1 strain (Fig. 1) is striking.

Morphological and genetic analysis carried out by Koide et al. (1998) indicated that the JF1 strain originated from the Japanese wild mouse, *M.musculusmolossinus*. Yoshiki and Moriwaki (2006) reported that the morphological and genetic characters of the JF1 strain are those of the *musculus* subspecies group.

The data of SNP-based genotyping suggest that the Japanese dancing mice from the ZMA described as *M.w.rotans* represent a crossbred, or derivatives thereof, between original Japanese waltzer of *M.musculusmolossinus* origin and European fancy or laboratory mice of *M.musculusdomesticus* origin. Most of their genome was replaced and occupied by *M.musculusdomesticus* type genome, probably through extensive breeding with European mice. The ZMA specimens have significant value to further elucidate the genetic status of the Japanese waltzer mice described in the old literature, and the origin of laboratory mice if their genome and morphology will be analysed in more detail, since it was reported that the JF1 ancestor is the origin of the *molossinus* genome in the classical inbred laboratory strains, contributing to the genetic diversity among the strains (Takada et al. 2013).

The house mouse has long been used in the laboratory and constitutes the “universal mammalian model” (Bonhomme 1986). However, the genealogy of laboratory strains and their relationships to one another and to wild forms is not yet completely clear. Nevertheless, it is critical to interpreting experimental results in laboratories and phylogenetic comparison between inbred strains and wild populations of *M.musculus* and other species (Carleton and Musser 2005).

## ﻿Conclusion

The knowledge of the whereabouts of the type specimens of the Japanese dancing mouse is of great importance not only in a historical perspective but also for the development of further studies to clarify the genetic background of laboratory mice.

The results of the present study indicate that the Japanese dancing mouse was derived from the Japanese house mouse before 1800 as a mutation with a characteristic black-and-white coat coloration and spinning behaviour. This mutation was maintained by inbreeding, first by mouse fanciers in Japan and in Europe and later in laboratories all over the world. The Japanese house mouse has been classified as *M.musculusmolossinus* and as *M.musculusmanchu*, but, in the latest classification (Carleton and Musser 2005), house mouse populations in Japan are considered to belong to either *M.musculusmusculus* or *M.musculuscastaneus*, or to a hybrid between these two subspecies.

The remarkable similarity between the coat colour of the type specimens of *M.wagnerirotans* and the specimens that constitute the JF1 strain, a laboratory mouse strain bred in the NIG, Japan, and thought to be derived from the Japanese house mouse based on genetic analysis, cannot be ignored. The variation of HB of the subjects also shows more similarity with the specimens described as *molossinus* by Schwarz and Schwarz (1943), than with other *Musmusculus* subspecies. Given the complexity of *Musmusculus*’ taxonomy and in particular the uncertainty of the origin of the Japanese house mouse (Nunome et al. 2010) caution should be taken when stating that the Japanese dancing mouse is derived from both *M.musculusmusculus* and *M.musculuscastaneus*.

## ﻿Additional information

### Author ORCIDs

Toyoyuki Takada https://orcid.org/0000-0001-6796-2085

Toshihiko Shiroishi https://orcid.org/0000-0003-0535-2016

Atsushi Yoshiki https://orcid.org/0000-0002-9450-5151

### Data availability

All of the data that support the findings of this study are available in the main text
